# Supplementary material for: Bacteria colonization in tumor microenvironment creates a favorable niche for immunogenic chemotherapy
Source: EMBO Mol Med. 2024 Jan 15;16(2):416–28. doi: 10.1038/s44321-023-00022-w (PMC10897177; doi:10.1038/s44321-023-00022-w)
Supplement: Supplementary file 1 — Appendix [file 44321_2023_22_MOESM1_ESM.pdf]

**Appendix (Supplementary Information)**

# Bacteria colonization in tumor microenvironment creates a favorable niche for immunogenic chemotherapy.

**See-Khai Lim[1], Wen-Ching Lin[1], Sin-Wei Huang[1], Yi-Chung Pan[1], Che-Wei Hu[1], Chung-Yuan Mou[2], Che-Ming Jack Hu[1]*, and Kurt Yun Mou[†]**

[1]Institute of Biomedical Sciences, Academia Sinica, Taipei, 11529, Taiwan.

[2]Department of Chemistry, National Taiwan University, Taipei, 106319, Taiwan.

[†]The author passed away on August 28th, 2023.

**[*]To whom correspondence should be addressed:**

Che-Ming Jack Hu, Ph.D.

Institute of Biomedical Sciences, Academia Sinica, Taipei, 11529, Taiwan.

Phone: +1-886-2-26523089

Fax: +1-886-2-26510774

Email: chu@ibms.sinica.edu.tw

## Table of content


| Content | Page |
|---|---|
| Appendix Figure S1. *In vivo* antitumor activity of 5-FU (IP) in combination with *E. coli* (DH5α; IT). | 3 |
| Appendix Figure S2. Body weight % of mice throughout their corresponding treatment period. | 3 |
| Appendix Figure S3. TILs analysis of MC38-syngeneic mice model received 5-FU based treatment. | 4 |
| Appendix Figure S4. Figures of tumor taken for each individual mice at the last day | 4 |
| Appendix Figure S5. *In vivo* antitumor activity of oxaliplatin in combination with *E. coli* (DH5α) on MC38 syngeneic models. | 5 |
| Appendix Figure S6: Gating strategy for tumor-infiltrating leukocyte analyses. | 6 |
| Appendix Figure S7. Tumor growth curve of mice treated with solvent (PBS), oxaliplatin (IP), *E. coli*-DH5α (IT) and their combinations on MC38 syngeneic murine model for tumor-infiltrating leukocytes analysis | 7 |
| Appendix Figure S8. Representative flow cytometry data for tumor-infiltrating leukocyte analysis (surface marker staining) | 8 |
| Appendix Figure S9. Representative flow cytometry Appendix Figures from tumor-infiltrating leukocyte analyses with intracellular staining of TNF-α. | 9 |
| Appendix Figure S10. Representative flow cytometry Appendix Figures from tumor-infiltrating leukocyte analyses with intracellular staining of IFN-γ. | 10 |
| Appendix Figure S11. Flow cytometry data presented in the form of cell count from tumor-infiltrating leukocyte analyses | 11 |
| Appendix Figure S12. Comparison of the effects of *in vitro* spleenocytes activation using live (no PS; MOE_L) versus growth-inhibited bacteria (with PS; MOE). | 12 |
| Appendix Figure S13. *In vitro* cytotoxicity of oxaliplatin on the MC38 cell line along with the individual or combination treatments of *E. coli* (E) and spleenocytes (S). | 13 |
| Appendix Figure S14. Images of MC38 cells co-cultured with spleenocytes (labelled with CFSE in green) and *E. coli* in the presence of Oxaliplatin. | 14 |
| Appendix Figure S15. Flow cytometry analysis of the viability of MC38-EGFP cells after 24 h co-culture with murine spleenocytes in various conditions. | 15 |
| Appendix Figure S16. Images of immunofluorescence staining to visualize the interactions between MC38-EGFP cells, spleenocytes, and *E. coli* in the presence of Oxaliplatin (2 µM). | 15 |

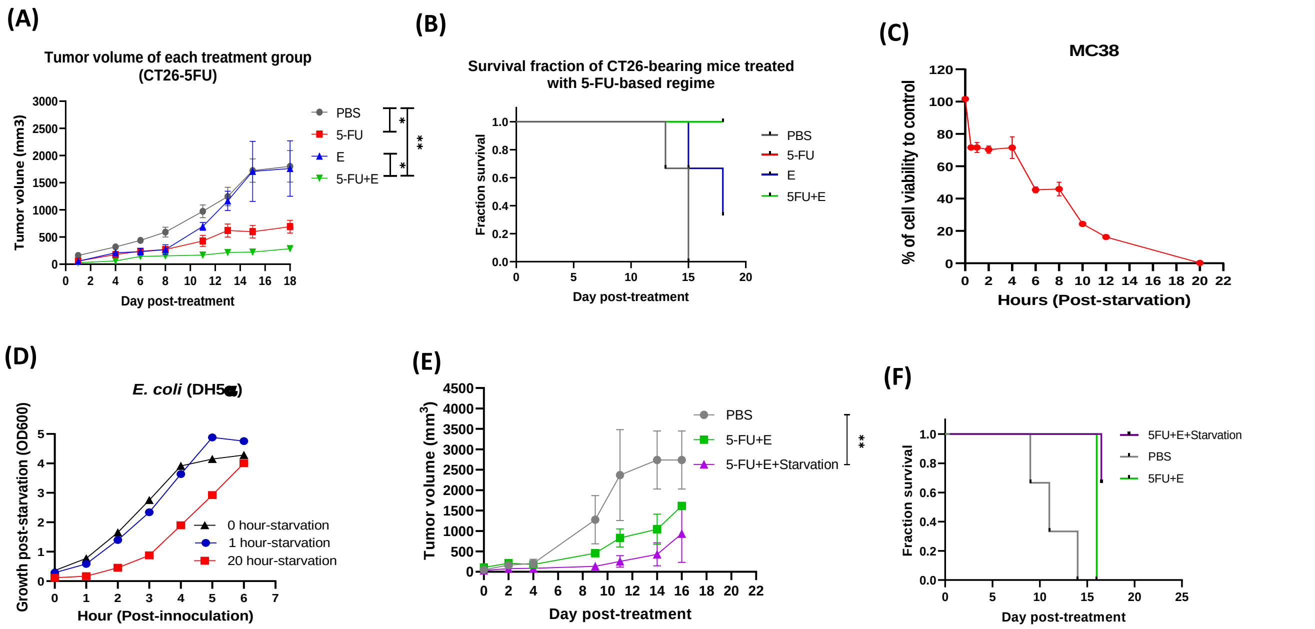


**Appendix Figure S1. *In vivo* antitumor activity of 5-FU (IP) in combination with *E. coli* (DH5α; IT) on Balb/C mice.** (A) Tumor growth curve for Balb/c mice inoculated with CT26 cells and (B) survival curve of Balb/c mice inoculated with CT26 cells that received different therapeutic interventions. **(**C) Viability of the MC38 cell line starved for various periods of time (n = 3). (D) Growth kinetics of *E. coli* (DH5α) after 1- and 20-hour starvation (n = 3). (E) *In vivo* anti-tumor activity of 5-FU+*E.* coli co-treatment with or without the intermittent fasting (4 non-consecutive 24-hour sessions in 2 weeks; n = 3) on MC38 syngeneic model. (F) Kaplan-Meier analysis of mouse survivals in (E).

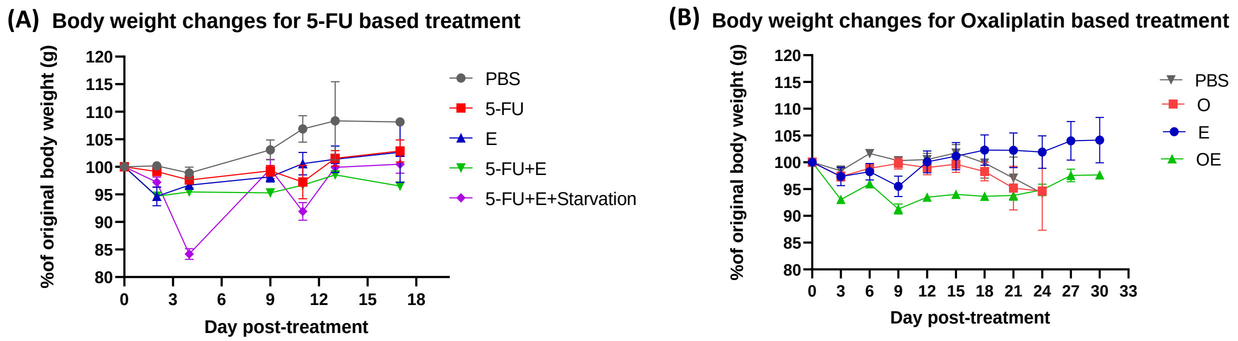


**Appendix Figure S2. Body weight % of mice throughout their corresponding treatment period (relative to body weight before initiation of treatment).** (A) Fluctuation of body weight for mice receiving solvent (PBS), 5-FU (IP), *E. coli* (IT), starvation or their combination as treatment. (B) Fluctuation of body weight for mice receiving solvent (PBS), oxaliplatin, *E. coli* or their combination as treatment.

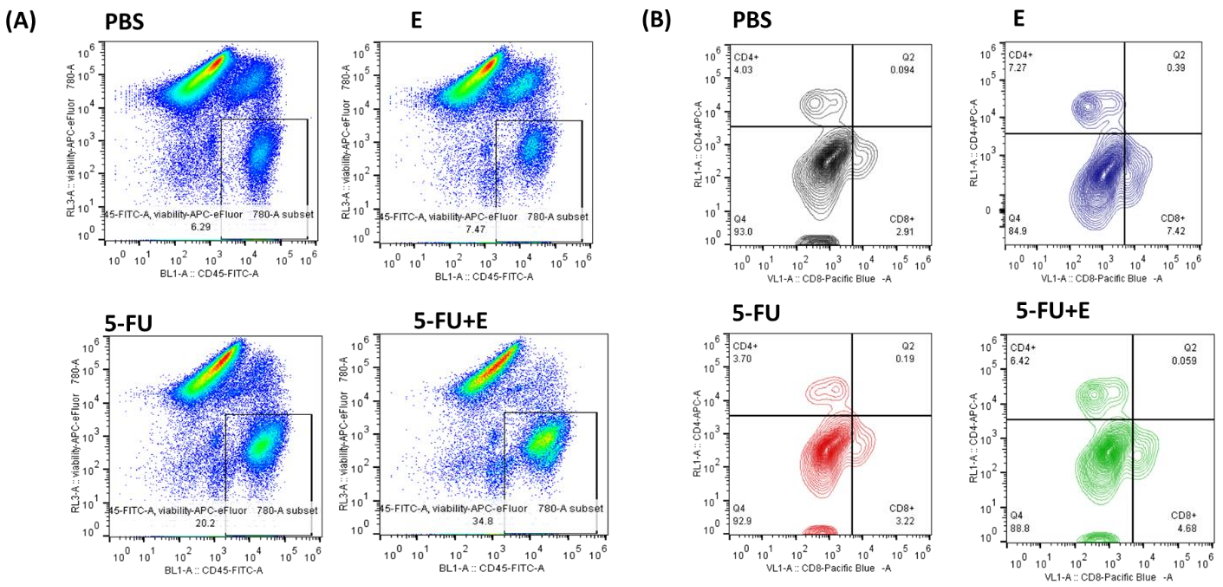


**Appendix Figure S3. TILs analysis of MC38-syngeneic mice model received 5-FU based treatment**. Staining of (A) viable CD45+ TILs (B) CD4+ and CD8+ TILs from tumors extracted from the 5-FU based treatment groups.

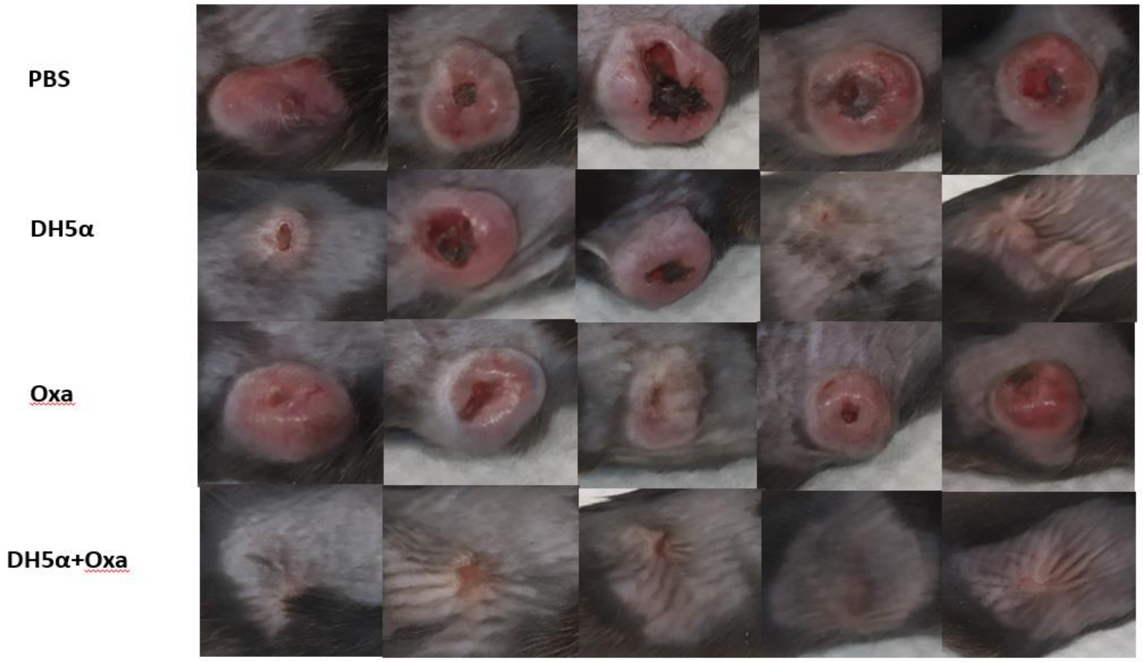


**Appendix Figure S4. Figures of tumor taken for each individual mice at the last day (18 days post-treatment) when all mice were considered alive.** Part of the figures were presented in Figure 2D.

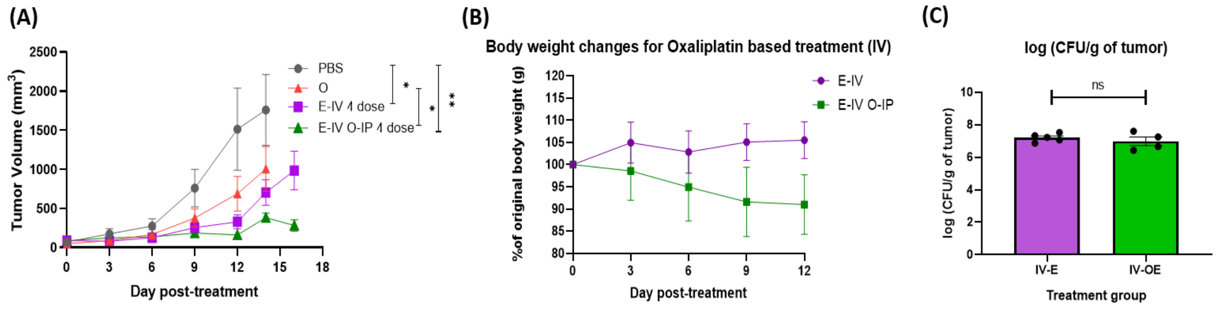


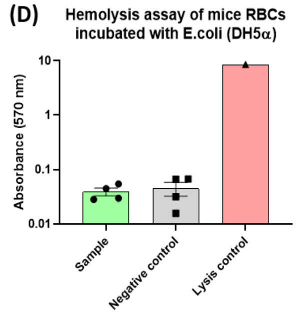


(E)

| | WBC | RBC | MCH | MCHC | %NEUT | %LYM | %MONO | %EOS | %BASO |
|---|---|---|---|---|---|---|---|---|---|
| **Unit:** | **x10$^3$ cells/μL** | **x10$^6$ cells/μL** | **pg** | **g/dL** | **%** | | | | |
| **D1** | 1.00 ± 0.15 | 10.41 ± 0.16 | 14.60 ± 0.06 | 30.32 ± 0.05 | 43.56 ± 6.80 | 44.36 ± 5.49 | 11.04 ± 2.24 | 1.04 ± 0.70 | 0.00 ±0.00 |
| **D3** | 17.87 ± 2.94 | 7.13 ± 0.15 | 14.46 ± 0.06 | 31.66 ± 0.14 | 39.66 ± 3.18 | 46.74 ± 4.12 | 12.50 ± 1.24 | 0.94 ± 0.10 | 0.16 ± 0.15 |
| **D7** | 8.93 ± 0.71 | 8.62 ± 0.16 | 14.32 ± 0.12 | 29.22 ± 0.24 | 23.04 ± 3.69 | 67.24 ± 5.77 | 7.28 ± 2.12 | 2.22 ± 0.24 | 0.22 ± 0.04 |
| **UT** | 10.09 ± 0.85 | 9.97 ± 0.12 | 14.78 ± 0.13 | 29.88 ± 0.24 | 26.20 ± 8.39 | 70.83 ± 7.97 | 1.35 ± 0.38 | 1.55 ± 0.21 | 0.08 ± 0.05 |

**Appendix Figure S5. *In vivo* antitumor activity of oxaliplatin in combination with *E. coli* (DH5α) on MC38 syngeneic models.** (A) *In vivo* antitumor activity of oxaliplatin (IP) in combination with *E. coli*-DH5α (IV) (B) Fluctuation of body weight for mice receiving *E. coli* (IV) or combination of oxaliplatin and *E. coli* (IV) as treatment. (C) Average colony forming unit (CFU) of bacteria extracted from homogenized tumor tissues of mice intravenously treated with *E. coli*. (D) Hemolysis assay using mice RBC and *E. coli*-DH5α. Briefly, mice blood was extracted via cardiac blood collection and RBC were separated via centrifugation followed by triple washing with PBS. The resulting RBC were diluted to a 1 % RBC suspension and incubated with 1 x 10$^6$ *E. coli* (Sample group) for 30 min at 37 °C. The samples were then centrifuged (350 xg for 5 min) and $A_{570}$ reading of the resulting supernatant (200 uL) were determined using a Tecan infinite microplate reader. A set of negative control (RBC incubated with PBS) and lysis control (incubated with 1 % TritonX-100) were included as a reference comparison for the assay. (E) Whole blood cell analyses for mice received IV-administration of *E. coli*. All mice received 1 dose (4 x 10$^8$ *E. coli* in PBS) of bacteria treatment and 100-200 uL of blood were collected via submandibular blood collection at day 1, 3 and 7 post-treatment. The samples were sent to National Laboratory Animal Center (Taipei, Taiwan) for whole blood cell count. A set of untreated mice (UT) were also included as control (n = 5).

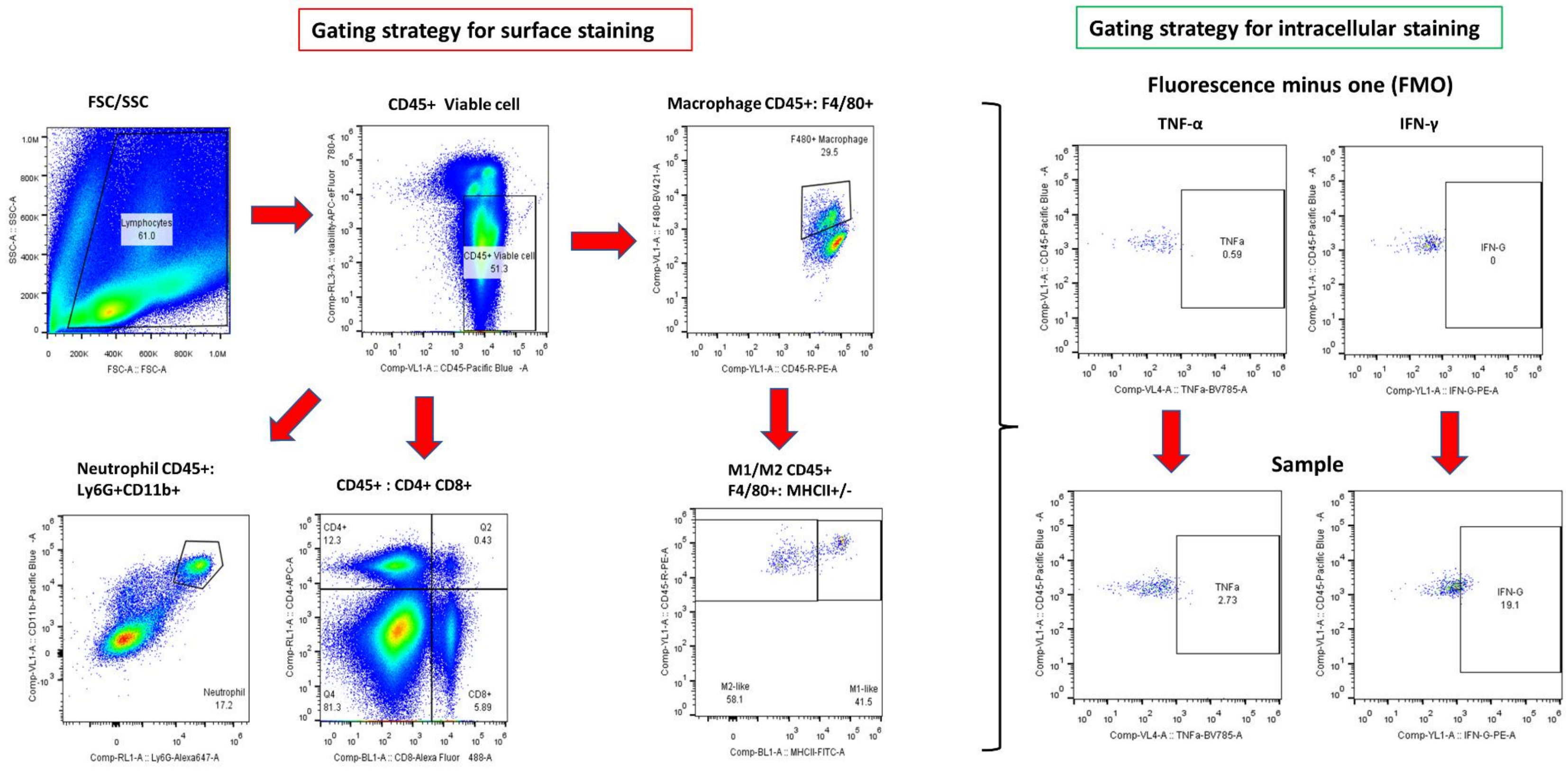


**Appendix Figure S6: Gating strategy for tumor-infiltrating leukocyte analyses.**

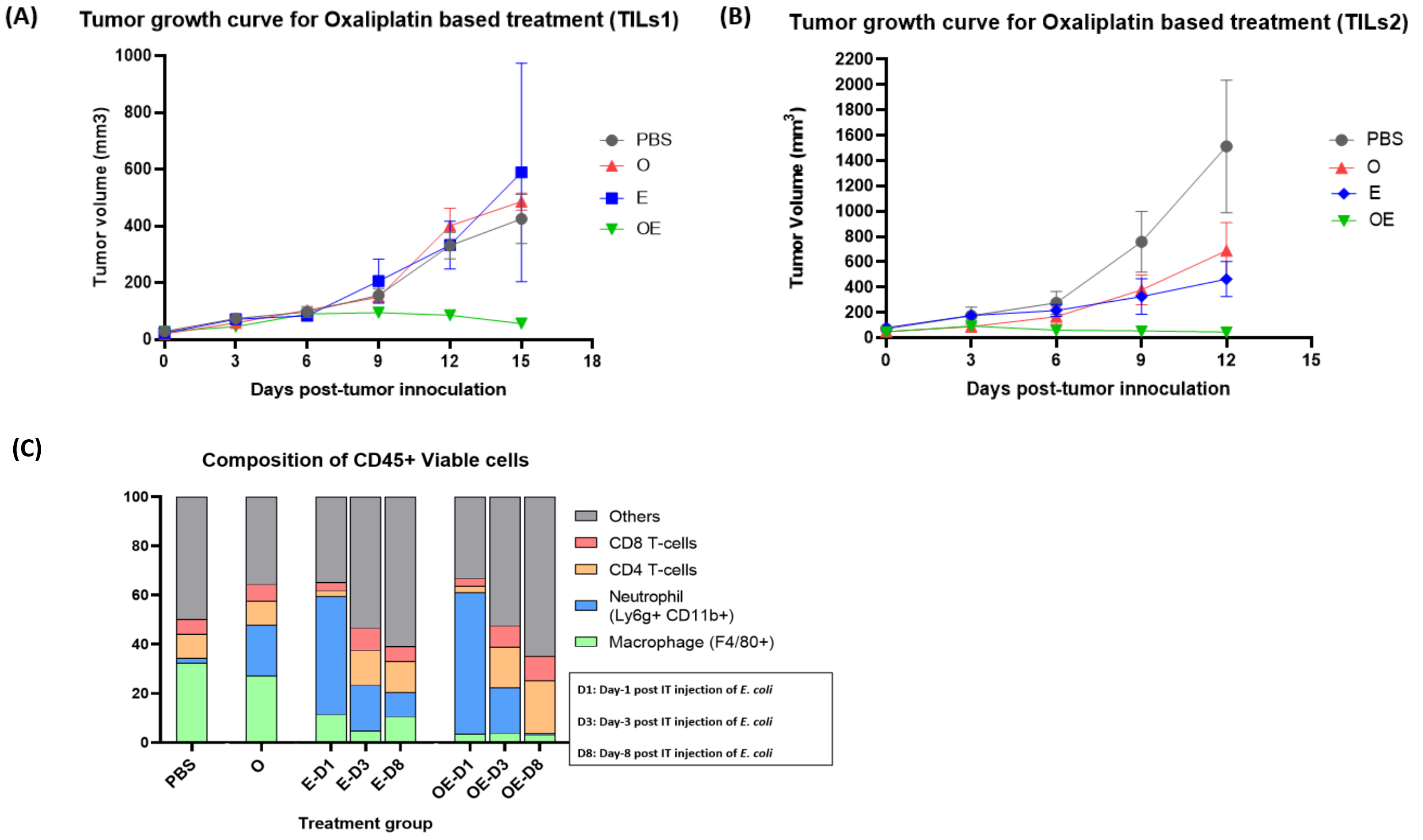


**Appendix Figure S7. Tumor growth curve of mice treated with solvent (PBS), oxaliplatin (IP), *E. coli*-DH5α (IT) and their combinations on MC38 syngeneic murine model for tumor-infiltrating leukocytes analysis** (A) surface staining of tumor-infiltrating immune cells and (B) intracellular staining of tumor-infiltrating immune cells. (C) Tumor infiltration leukocytes profile for various time points (1, 3 and 8 days) after receiving the last treatment of *E. coli*. *E. coli* was administered intratumorally while oxaliplatin were administered intraperitoneally.

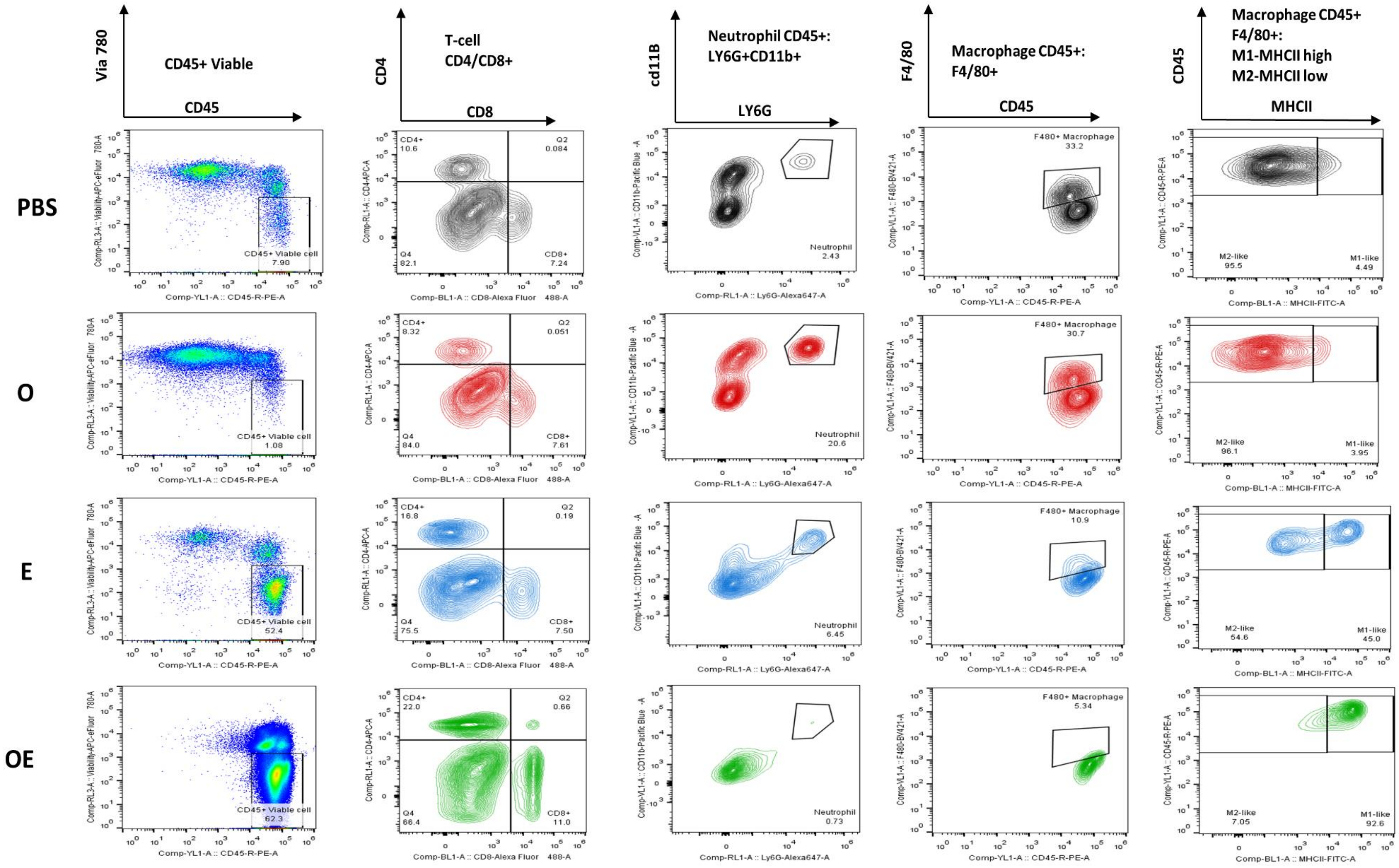


**Appendix Figure S8. Representative flow cytometry data for tumor-infiltrating leukocyte analysis (surface marker staining)**

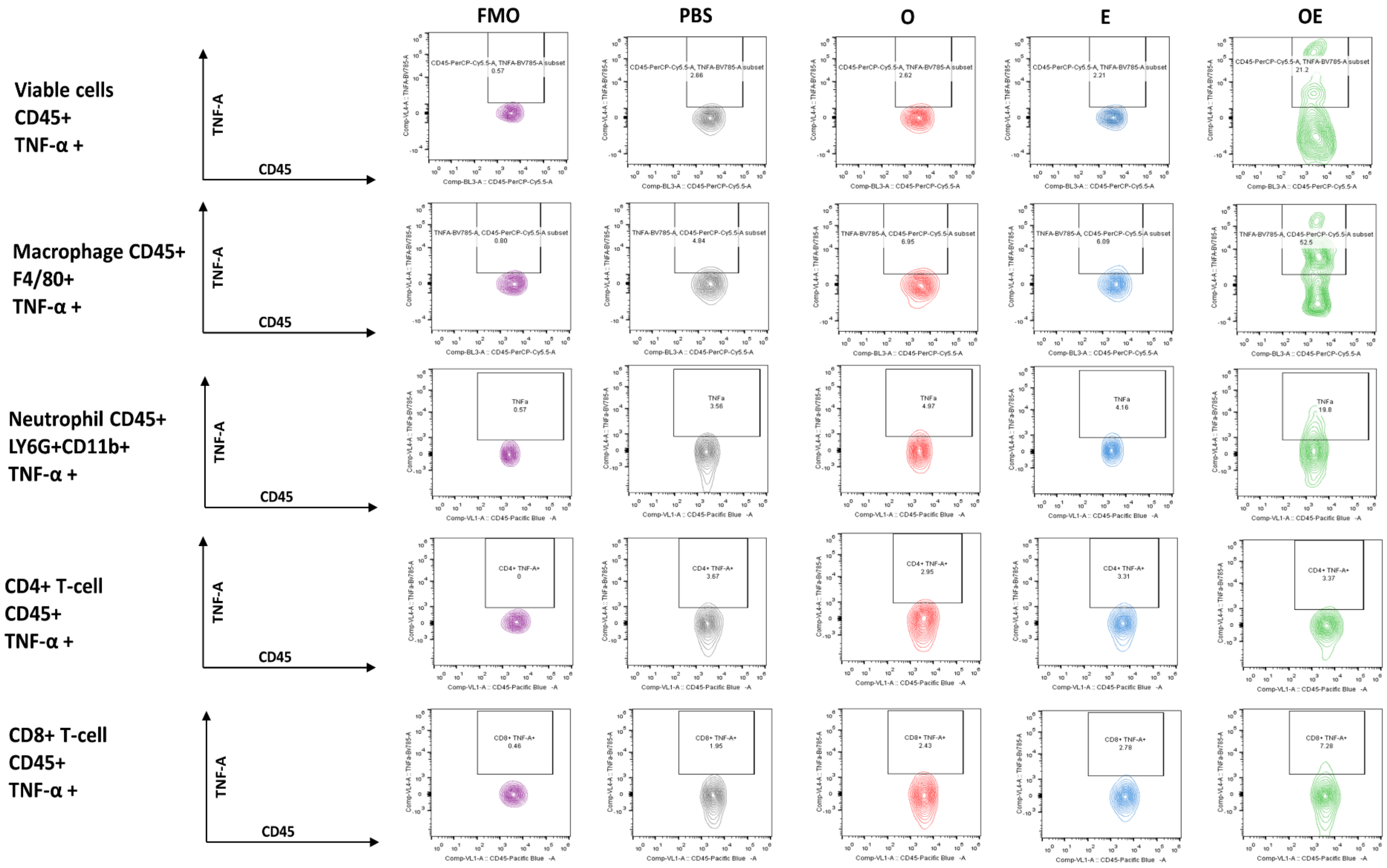


**Appendix Figure S9. Representative flow cytometry Appendix Figures from tumor-infiltrating leukocyte analyses with intracellular staining of TNF-α.**

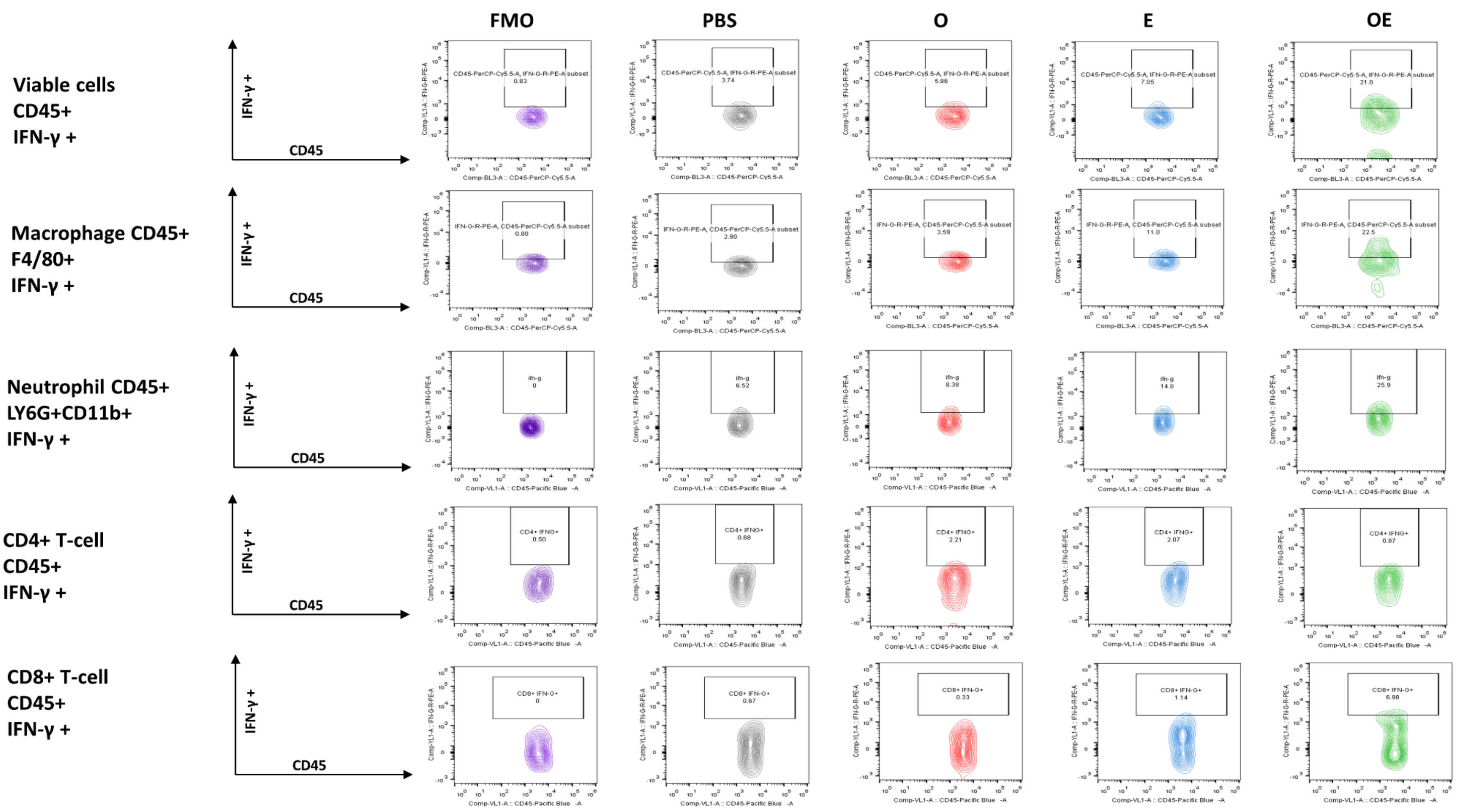


**Appendix Figure S10. Representative flow cytometry Appendix Figures from tumor-infiltrating leukocyte analyses with intracellular staining of IFN-γ.**

(A)

| Cell/g of tumor | CD45+ Viable cell | CD4+ T cell | CD8+ T cell | Neutrophil | Macrophage | M1-like Macrophage |
|---|---|---|---|---|---|---|
| PBS1 | 3.53E+03 | 9.95E+02 | 4.89E+02 | 2.72E+02 | 1.17E+03 | 5.25E+01 |
| PBS2 | 1.79E+04 | 9.05E+02 | 6.19E+02 | 1.12E+02 | 4.80E+03 | 1.13E+03 |
| PBS3 | 5.12E+03 | 1.72E+02 | 6.84E+01 | 5.89E+01 | 1.56E+03 | 2.92E+02 |
| O1 | 6.25E+03 | 3.85E+02 | 3.52E+02 | 1.06E+03 | 8.38E+02 | 1.76E+02 |
| O2 | 6.74E+03 | 7.67E+02 | 5.62E+02 | 1.55E+03 | 1.74E+03 | 3.16E+02 |
| O3 | 2.35E+03 | 3.62E+03 | 1.36E+03 | 6.28E+03 | 7.22E+02 | 2.85E+01 |
| E1 | 1.24E+06 | 3.86E+05 | 1.85E+05 | 1.46E+05 | 1.96E+05 | 1.92E+05 |
| E2 | 6.54E+04 | 1.32E+04 | 5.91E+03 | 7.51E+03 | 7.10E+03 | 3.20E+03 |
| E3 | 9.72E+03 | 1.71E+03 | 9.08E+02 | 3.60E+03 | 1.02E+03 | 8.33E+02 |
| OE1 | 2.08E+06 | 8.81E+05 | 4.41E+05 | 4.36E+03 | 1.03E+05 | 9.42E+04 |
| OE2 | 1.39E+06 | 4.85E+05 | 2.73E+05 | 1.99E+04 | 7.42E+04 | 6.87E+04 |
| OE3 | 2.71E+06 | 1.65E+06 | 6.32E+05 | 1.99E+04 | 1.67E+05 | 1.57E+05 |

(B)

| Cell/g of tumor | IFN-g+ Macrophage | TNF-a+ Macrophage | IFN-g+ Neutrophil | TNF-a+ Neutrophil | IFN-g+ CD8 T cells | TNF-a+ CD8+ T-cells | TNF-a+ CD45+ cells | IFN-g+ CD45+ cells |
|---|---|---|---|---|---|---|---|---|
| PBS1 | 1.81E+01 | 3.46E+01 | 9.89E+00 | 7.14E+00 | 9.34E+00 | 3.24E+01 | 1.10E+02 | 2.81E+02 |
| PBS2 | 4.32E+01 | 5.59E+01 | 2.33E+01 | 9.41E+00 | 6.09E+00 | 6.09E+00 | 1.57E+02 | 1.32E+02 |
| O1 | 8.26E+01 | 1.08E+02 | 3.85E+01 | 6.42E+01 | 7.34E+00 | 2.02E+01 | 2.55E+02 | 2.68E+02 |
| O2 | 6.17E+01 | 5.81E+01 | 3.87E+01 | 2.30E+01 | 2.30E+01 | 3.51E+01 | 2.45E+02 | 1.89E+02 |
| E1 | 4.00E+02 | 1.68E+02 | 5.12E+02 | 8.96E+01 | 4.02E+01 | 7.31E+01 | 2.07E+03 | 1.07E+03 |
| E2 | 1.74E+03 | 5.43E+02 | 6.63E+02 | 2.51E+02 | 1.12E+02 | 1.84E+02 | 4.07E+03 | 2.35E+03 |
| OE1 | 3.00E+03 | 1.17E+04 | 1.29E+03 | 8.78E+02 | 9.27E+02 | 1.83E+03 | 4.12E+03 | 9.12E+03 |
| OE2 | 1.29E+03 | 2.46E+03 | 5.71E+02 | 4.86E+02 | 1.00E+03 | 6.57E+02 | 1.77E+03 | 3.74E+03 |

**Appendix Figure S11. Flow cytometry data presented in the form of cell count from tumor-infiltrating leukocyte analyses (A) Data of surface markers staining of tumor-infiltrating leukocytes. (B) Data of intracellular staining of TNF-α and IFN-γ of tumor-infiltrating leukocytes.**

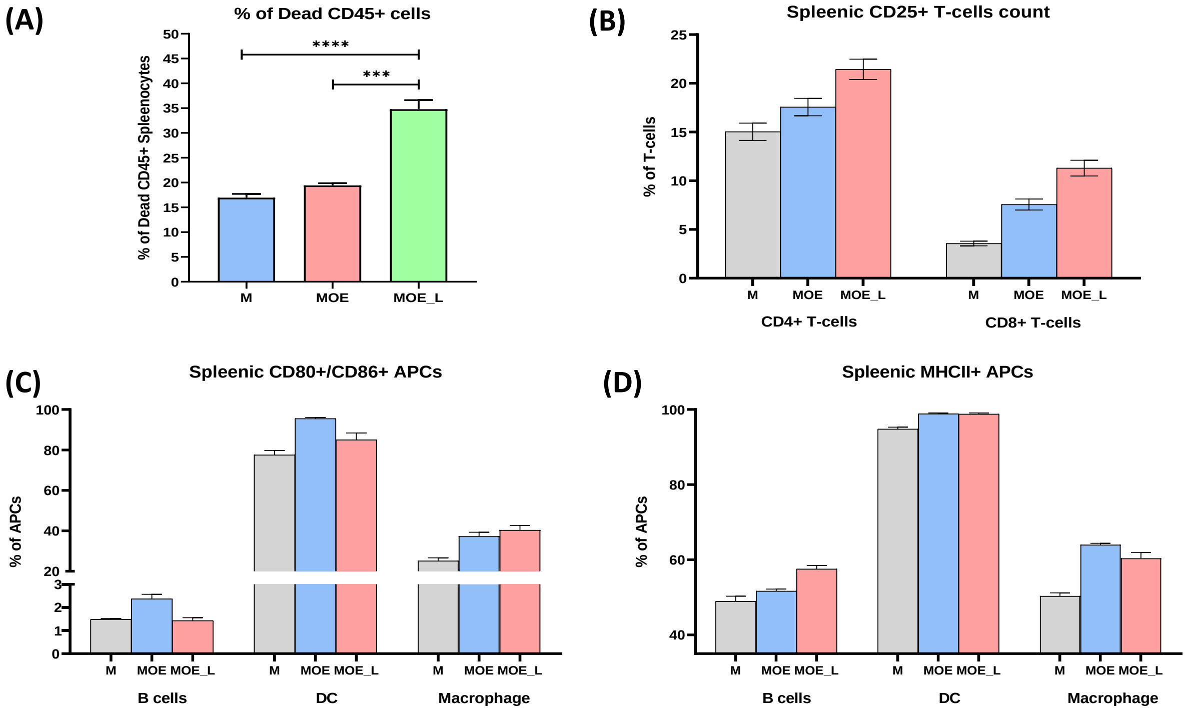


**Appendix Figure S12. Comparison of the effects of *in vitro* spleenocytes activation using live (no PS; MOE_L) versus growth-inhibited bacteria (with PS; MOE).** (A) Percentage of dead spleenocytes 24 hours post-treatment. (B) Percentage of activated T-cells (CD25+) 24 hours post-treatment. (C) Percentage of CD80+/86+ population for each type of APCs at 24 hours post-treatment. (D) Percentage of MHCII+ population for each type of APCs at 24 hours post-treatment.

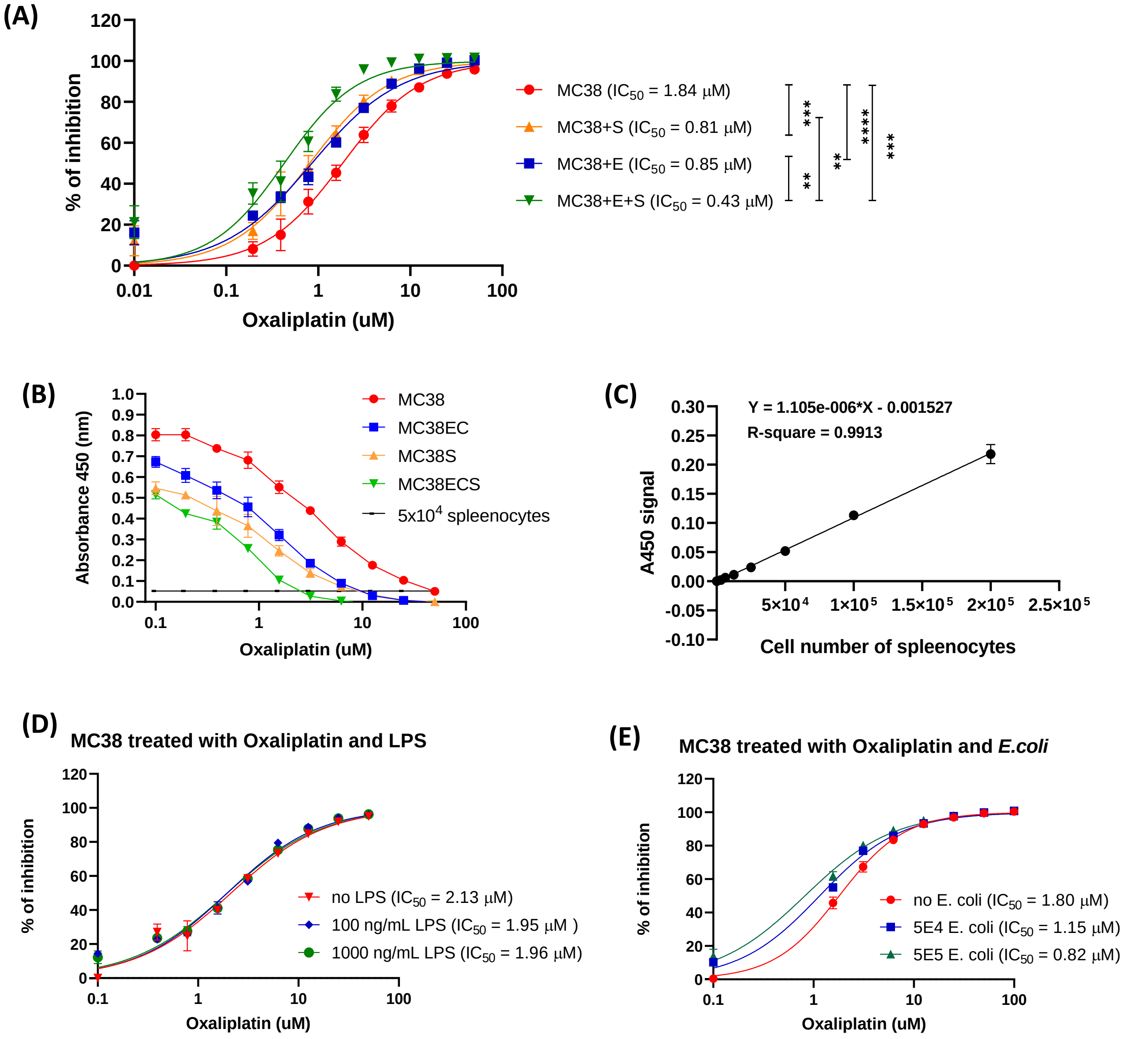


**Appendix Figure S13.** (A) *In vitro* cytotoxicity of oxaliplatin on the MC38 cell line along with the individual or combination treatments of *E. coli* (E) and spleenocytes (S). (B) CCK-8 signal readings (A450) of the cytotoxicity assay performed in (A). The CCK-8 responsivity of 5 x $10^4$ spleenocytes (amount seeded in the co-culture assay) were presented as black line. (C) Standard curve demonstrating correlation between spleenocyte counts with the CCK-8 signals (A450). Spleenocytes were counted using hemocytometer under microscope prior to the generation of standard curve. (D) MC38 treated with various concentration of oxaliplatin in the presence of 100 ng/mL or 1000 ng/mL LPS (without spleenocytes). (E) MC38 treated with various concentrations of oxaliplatin in the presence of 5E4 and 5E5 *E. coli* (without spleenocytes).

(A)

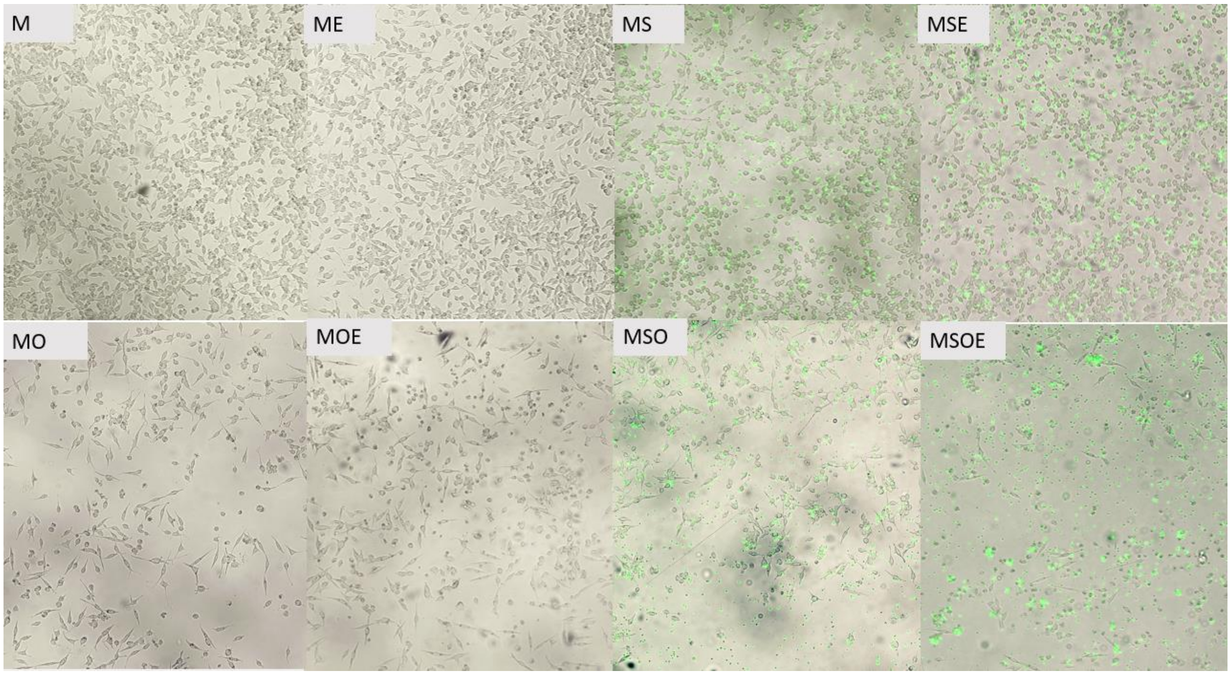


(B)

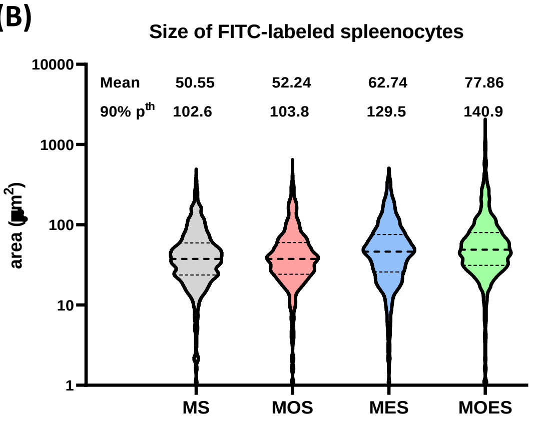


**Appendix Figure S14. (A) Images of MC38 cells co-cultured with spleenocytes (labelled with CFSE in green) and *E. coli* in the presence of Oxaliplatin (1.56 µM).** The cells were incubated with the corresponding treatment for 48 hours and were gently washed twice with warm PBS to remove non-adherent spleenocytes before observation. (B) Distribution of the size of spleenocyte clusters (area; um$^2$) determined from each treatment group.

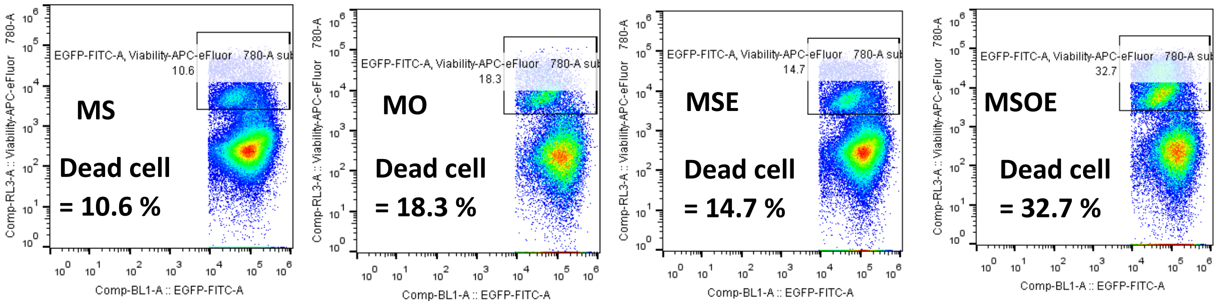


**Appendix Figure S15. Flow cytometry analysis of the viability of MC38-EGFP cells after 24 h co-culture with murine spleenocytes in various conditions. Viability of MC38-EGFP cells were determine using Efluor780 fixable viability dye (Thermofisher Scientific).** (M: MC38-spleenocytes; MO: MC38-spleenocytes with 2 uM oxaliplatin; ME: MC38-spleenocytes with 2E6 *E. coli*; MOE: MC38-spleenocytes with 2 uM oxaliplatin and 2E6 *E. coli*)

**Oxaliplatin with *E. coli***

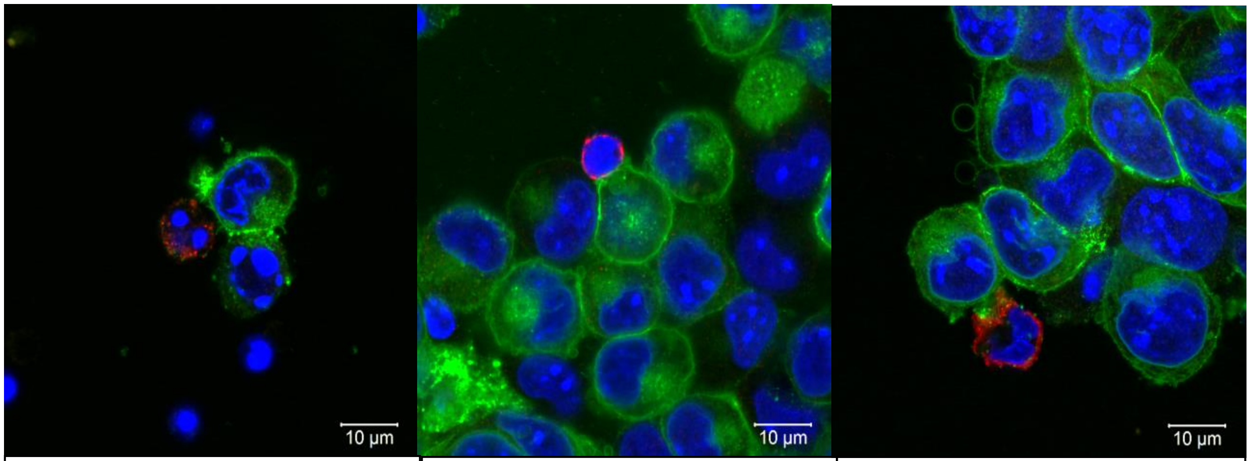


| | | |
|---|---|---|
| • **Red: CD11c -DC**<br>• **Green: anti-EGFP (MC38)**<br>• **Blue: Hoechst** | • **Red: CD8+ T-cell**<br>• **Green: anti-EGFP (MC38)**<br>• **Blue: Hoechst** | • **Red: F4/80+ Macrophage**<br>• **Green: anti-EGFP (MC38)**<br>• **Blue: Hoechst** |

**Appendix Figure S16. Images of immunofluorescence staining to visualize the interactions between MC38-EGFP cells, spleenocytes, and *E. coli* in the presence of Oxaliplatin (2 µM).** The co-culture was first washed, fixed, blocked, and stained with Alexa Fluor® 488 anti-GFP (1:50; Biolegend: 338008) to highlight MC38-EGFP cells. To identify specific immune cell types binding to MC38-EGFP cells, we individually stained the samples with rabbit anti-mouse CD8a (1:50 Abcam: ab217344), rabbit anti-mouse CD11c (1:50 Abcam: ab219799), and rabbit anti-mouse F4/80 (1:50 Abcam: ab300421). These primary antibodies were incubated overnight, followed by staining with secondary antibodies (1:2000; Goat anti-rabbit CF-568 Biotum 20102) and Hoechst 33342 (1:10000; Invitrogen) for an hour before visualization using confocal microscopy (ZEISS Airyscan; LSM880). It's worth noting that we attempted to stain CD4-T cells and B-lymphocytes using anti-mouse CD4 Alexa 647 (1:50; BD Pharmingen 557681) and anti-mouse CD19 Alexa 647 (1:50; BD Pharmingen 557684), but no binding of these cell types to the MC38-EGFP cells was observed in the samples.
